# The emergency use authorization for COVID‐19 convalescent plasma reduced mortality

**DOI:** 10.1002/hsr2.1463

**Published:** 2023-08-06

**Authors:** Nigel Paneth, Arturo Casadevall, Michael Joyner

**Affiliations:** ^1^ College of Human Medicine Michigan State University East Lansing Michigan USA; ^2^ Department of Molecular Microbiology & Immunology The Johns Hopkins University Baltimore Maryland USA; ^3^ Department of Anesthesiology The Mayo Clinic Rochester Minnesota USA

To the Editor,

In their review of the evidence base for emergency use authorizations (EUAs) by the US Food and Drug Administration (FDA),^1^ the authors assert that “*The presence of EUAs may discourage participation in relevant clinical trials*.” But the data in the paper suggest otherwise. Tables 2 and 3 show that for the eight agents examined, more than four times as many randomized controlled trials (RCTs) were conducted between the time the EUAs were issued and September 2021 than before the EUAs. In the case of convalescent plasma, the ratio of post‐EUA to pre‐EUA RCTs was 9 to 1 (18 vs. 2). Rather than suggesting that EUAs inhibited the development of RCTs, the data indicate that EUAs encouraged the performance of RCTs, perhaps by drawing attention to promising interventions that needed stronger scientific support.

In discussing the two RCTs that preceded the EUA for convalescent plasma (see fig. 1), the authors state that “*neither of these showed a statistically significant treatment effect*.” But Li et al. reported that among participants with severe disease, clinical improvement defined as patient discharge or a reduction of two points on a six‐point disease severity scale—occurred in 91.3% (21/23) of the convalescent plasma group, but in just 68.2% (15/22) of the control group (*p* = 0.03).^2^ Patients with life‐threatening disease showed no benefit.

Both trials showed overall lower mortality in their CCP arms. Li et al. reported eight deaths in the treatment arm, 12 deaths in controls, while Gharbaran et al. found six deaths in CCP treated, 11 in controls. Both trials were terminated early and were underpowered, but if combined, overall mortality in CCP‐treated participants was 14 of 94, compared to 23 of 93 in controls, a 40% reduction, which, while not quite significant at the 0.05 level (*p* = 0.09), cannot be dismissed in the face of an epidemic.

Furthermore, the authors make no mention of findings of the Expanded Access Program (EAP), initiated 5 months before the EUA for CPP. This observational study of more than 94,000 recipients of convalescent plasma provided unequivocal evidence that CCP was safe at a time when many were concerned about antibody‐dependent enhancement and the possibility that the administration of antibody would trigger cytokine storms.

Even more significantly, data from the EAP led directly to the FDA decision to issue an EUA for CPP. The EAP asked whether the level of antibody in the administered plasma, all other factors considered, bore a relationship to mortality in recipients. Joyner et al.^3^ found that, in some 3000 participants in whom antibody was measured in residual samples of the transfused plasma, a stepwise gradient of decreasing mortality was seen in recipients as level of antibody in the plasma increased. The FDA then performed its own analyses of residual samples from the EAP program, using a different antibody test, and drew nearly identical conclusions. Bias is very unlikely since there was no way of knowing, at the time of treatment, anything about the antibody content of the transfused plasma. The demonstration of a dose–response effect for antibody dose and mortality is powerful evidence for the efficacy of CCP.

Most importantly, especially in light of the later RECOVERY trial, both of these findings were restricted to patients treated early. In the Joyner et al analysis, mortality benefit was seen in patients not mechanically ventilated and in the FDA analysis mortality was lower in high‐titer CCP recipients who were unventilated, under age 80 and treated within 3 days of symptom onset. In both analyses high‐titer plasma was associated with a mortality reduction of more than one‐third compared to low‐titer plasma in these categories of patients.

The authors describe the RECOVERY CCP trial as “robust” and lament that it was not adopted more widely in other countries. Unfortunately, RECOVERY cannot be described as robust. Not only did the CCP treatment arm in RECOVERY include 494 participants who did not actually receive CCP, but RECOVERY was conducted in an inpatient population whose overall mortality rate was 24% and who had experienced COVID symptoms for up to 14 days. RECOVERY was several times the size of the next largest trial of CCP, but sample size cannot substitute for biological incoherence. The convalescent serum literature, which dates back to the 1890s, is replete with the insistence that passive antibody therapy is only useful early in the course of the disease. A reanalysis of RECOVERY data showed that participants treated in the first 7 days and who had yet to mount an antibody response were likely to have benefited from CCP.^4^

In fact, the RECOVERY result was very influential but in a negative way. In the United States, in fall of 2020 and early winter of 2021, our published analysis shows that some 40% of inpatients in the United States were receiving CPP. By March 2021, after publication of RECOVERY, the use of CPP in US hospitals had dropped to 10%. Unfortunately, this decline in use was powerfully correlated with an increase in COVID mortality.^5^

The value of early use of CPP has been demonstrated in two carefully conducted outpatient RCT's which documented large reductions in respiratory deterioration and hospitalization, respectively, one of which (Libster et al.) is cited in fig. 1, and the other, by Sullivan et al. appeared after September 2021, the end of the review period.^6^ The most recent overview of all studies in the field show that mortality is reduced by some 37% with early use in hospitalized patients,^7^ and is especially valuable in immunocompromised patients.

We submit that the FDA ruled correctly in providing an EUA for CPP. The EUA allowed US COVID‐19 patients to receive a therapy that historical data indicated was likely to be effective, and which was subsequently shown to be clearly effective against COVID‐19 when properly used. Many Americans are alive today because of the CCP EUA. How many more lives might have been saved over the last 2 years if CCP use had not declined based on RCTs that tested the wrong use case?

## AUTHOR CONTRIBUTIONS

**Nigel Paneth**: Conceptualization; methodology; writing—original draft. **Arturo Casadevall**: Conceptualization; methodology. **Michael Joyner**: Conceptualization; methodology; writing—review & editing.

## CONFLICT OF INTEREST STATEMENT

The authors declare no conflict of interest.

## Data Availability

Data sharing not applicable to this article as no datasets were generated or analyzed during the current study.
